# Regulatory Role of Nrf2 Signaling Pathway in Wound Healing Process

**DOI:** 10.3390/molecules26092424

**Published:** 2021-04-21

**Authors:** Ipek Süntar, Sümeyra Çetinkaya, Emiliano Panieri, Sarmistha Saha, Brigitta Buttari, Elisabetta Profumo, Luciano Saso

**Affiliations:** 1 Department of Pharmacognosy, Faculty of Pharmacy, Gazi University, Etiler, Ankara 06330, Turkey; 2 Biotechnology Research Center of Ministry of Agriculture and Forestry, Yenimahalle, Ankara 06330, Turkey; cetinkayasumeyra0@gmail.com; 3 Department of Physiology and Pharmacology “Vittorio Erspamer”, La Sapienza University, 00185 Rome, Italy; emiliano.panieri@hotmail.it (E.P.); luciano.saso@uniroma1.it (L.S.); 4 Department of Cardiovascular and Endocrine-Metabolic Diseases, and Aging, Italian National Institute of Health, 00161 Rome, Italy; sarmistha_pharmacol@yahoo.com (S.S.); brigitta.buttari@iss.it (B.B.); elisabetta.profumo@iss.it (E.P.)

**Keywords:** Nrf2, wound healing, skin, inflammation, antioxidant

## Abstract

Wound healing involves a series of cellular events in damaged cells and tissues initiated with hemostasis and finally culminating with the formation of a fibrin clot. However, delay in the normal wound healing process during pathological conditions due to reactive oxygen species, inflammation and immune suppression at the wound site represents a medical challenge. So far, many therapeutic strategies have been developed to improve cellular homeostasis and chronic wounds in order to accelerate wound repair. In this context, the role of Nuclear factor erythroid 2-related factor 2 (Nrf2) during the wound healing process has been a stimulating research topic for therapeutic perspectives. Nrf2 is the main regulator of intracellular redox homeostasis. It increases cytoprotective gene expression and the antioxidant capacity of mammalian cells. It has been reported that some bioactive compounds attenuate cellular stress and thus accelerate cell proliferation, neovascularization and repair of damaged tissues by promoting Nrf2 activation. This review highlights the importance of the Nrf2 signaling pathway in wound healing strategies and the role of bioactive compounds that support wound repair through the modulation of this crucial transcription factor.

## 1. Introduction

The skin acts as a barrier maintaining skin integrity and homeostasis against harmful pathogens and physical stressors. Wound healing is a complex and well-organized multi-step process that takes place with the participation of cytokines, growth factors and matrix metalloproteinases (MMPs). It consists of a series of multiple stages, mainly hemostasis, inflammation, proliferation and remodeling. The process of physiological hemostasis includes blood coagulation in the wound area and ends with the formation of a hemostatic plug. Thereafter, the inflammation, serves different functions in the wound area, since it initiates thrombocyte degranulation and promotes the recruitment of neutrophils and monocytes for the removal of bacteria, dead cells and tissue debris and tissue repair. The cell migration process activated by fibroblast growth factor (FGF) and transforming growth factor (TGF) is a fundamental step that precedes the subsequent formation of granulation tissue and extracellular matrix (ECM), which in turn induces proliferation and tissue remodeling [1].

The excessive production or inefficient detoxification of reactive oxygen species (ROS), crucial regulators of the wound healing process, can potentially inflict oxidative damage. Importantly, this is regarded as the main cause of chronic wounds [2]. In chronic wounds (such as diabetic foot ulcers, venous ulcers, bed sores), the healing time is slower due to several reasons such as the absence of ECM proteins, increased ROS production, deterioration of neovascularization, hypoxia, decreased collagen accumulation and reduced secretion of angiogenic growth factors [3,4]. Moreover, the imbalance between the production and degradation of growth factors by MMPs further contributes to prolonged the healing time. MMPs are endopeptidases that have the capacity to degrade all the components of the ECM. MMPs are secreted as proenzymes by many cell types, including lymphocytes and granulocytes, but in particular by activating macrophages [5]. MMPs are regulated by a family of inhibitors called the tissue inhibitors of matrix metalloproteinases (TIMPs), which are constitutively produced by a variety of cells. Changes in actual MMP activity are thus dependent on the balance between production and activation of MMPs and the local levels of TIMPs. In addition to the remodeling of the ECM as a result of increased MMP activity during wound healing, MMPs also regulates the cell–cell and cell–matrix signal by the release of various cytokines and growth factors in the ECM [6,7,8,9]. MMPs are crucial in all stages of wound healing as, in addition to the ECM remodeling, they also promote keratinocytes migration and re-epithelization [10]. In diabetic wounds, TIMP-1 and TIMP-2 mRNA and protein levels decrease while MMP-2 and MMP-9 mRNA and protein levels increase when compared to normal wounds [11]. This indicates that the balance between MMP and TIMP is maintained during the normal wound healing process while its disruption can lead to pathological events.

The initial stage in fighting invading pathogens and stimulating cellular signal transduction pathways in response to skin damage begins with the increased ROS production [12]. In response to an excessive ROS production within injured and inflamed tissue, Nuclear factor E2 p45-related factor 2 (Nrf2) is synthesized to support wound repair [1]. This suggests that the amount of ROS secreted from epithelial cells acts as a sensor for Nrf2 expression. In addition, ROS accumulation is required to prevent infections in the wound area [13]. Apart from ROS, nitric oxide (NO) as well as hydrogen sulfide (H2S)-driven signaling can also play an important role through the modulation of the Nrf2 transcription factor [14].

Nrf2/Kelch-like ECH-associated protein 1 (Nrf2/Keap1) is a crucial signaling pathway that protects against environmental stressors such as ROS, electrophilic and proteotoxic stress [15]. Nrf2 plays a key role in reducing cellular stress and restores redox homeostasis by regulating the expression of more than 1000 genes such as heme oxygenase 1 (HMOX1), thioredoxin (TRX), glutathione reductase (GSR), glutamate-cysteine ligase catalytic subunit (Gclc) and modifier subunit (Gclm), superoxide dismutase 1 (SOD1) and catalase [16,17]. In addition, Nrf2 signaling also takes part in the regulation of the cellular response to inflammation by inhibiting inflammation via suppression of pro-inflammatory cytokines and controls fundamental cellular processes such as apoptosis, autophagy, angiogenesis, proliferation and cell migration [18,19]. Antioxidant enzymes, phase II and III detoxification enzymes, proteases, chaperones, inflammatory factors and growth factors are among the Nrf2 targets [20,21,22]. Among the Nrf2 activators, NO, small interfering RNA (siRNA), bee venom, as well as the administration of various active compounds have been reported as effective therapeutic approaches to promote wound healing [12].

Apart from Nrf2, signal transducer and activator of transcription 3 (STAT3) (in proliferation and differentiation), Smad proteins (in collagen expression, acceleration of wound healing) and Forkhead box protein N1 (FOXN1) (in re-epithelization) are other transcriptional regulators involved in wound healing. The transcription factor p53 plays a crucial role in cutaneous wound healing process [23,24]. It has been reported that p53, one of the most well-known tumor suppressor genes, also mediates the Nrf2 response. The role of p53 is dual. It acts as an antioxidant in moderate oxidative stress conditions to support cell survival. In contrast, it shows pro-oxidant activity under conditions associated with increasing cellular stress and triggers apoptosis. Therefore, p53 is involved in the expression of Nrf2 mediated antioxidant genes [4].

Therefore, the present review will examine the structure of the Nrf2 transcription factor and its functional interactions and activation. Furthermore, the relationship between Nrf2 and wound healing as well as the consequences of Nrf2 induction in diabetic wound healing will be discussed in light of the most recent findings obtained from in vitro and in vivo studies.

## 2. Wound Healing Process

Wound healing begins immediately after wound formation and developed in three distinct phases, each of them associated with complex and diverse processes including hemostasis, inflammation, proliferation and maturation. In the hemostasis phase, the wound starts to be closed by clotting. Hemostasis begins just after blood leaks out of the body. At first, blood vessels constrict in order to restrict the blood flow. Then, the platelets coagulate to form a plug, while subsequently, a number of fibrin strands start to adhere and promote the formation of a thrombus, which entraps the blood cells in the wounded area. The second stage of healing is the inflammatory process, which starts immediately after blood vessels, and is characterized by an intense chemotaxis promoted by the leakage of a transudate that attracts the repair cells to the wounded site. This natural phase of the healing response is finalized to control bleeding and prevent infection by removal of damaged cells and pathogens. In this stage, the typical signs of the inflammatory response known as heat, pain and redness, occur. While regarded as part of the physiological wound healing process, this phase can become detrimental if it prolongs or proceeds with excessive magnitude. In the proliferative phase, characterized by the formation of granulation tissue, new blood vessels and extensive re-epithelialization, the wound is rebuilt with extracellular matrix and collagen laid down by the fibroblasts, and then it begins to contract as a consequence of myofibroblasts activity. The granulation tissue is formed to provide sufficient oxygen and nutrients to achieve the optimal regeneration of the damaged tissue [25,26]. Afterwards, the re-epithelialization characterized by the intense proliferation and migration of keratinocytes towards the injury, progressively begins to resurface the lesion and concludes the proliferative phase of the wound process. The subsequent step, known as the maturation stage, is essentially a remodeling phase of the tissue wherein all the events activated in the earlier stages are markedly attenuated. In this stage, type III collagen is gradually replaced by type I collagen and the wound closes completely due to the crosslinking formation between the aligned collagen fibers. As a result, the scar thickness is progressively reduced whilst the tensile strength of the tissue is increased. 

Given the complexity and the need for a sequential coordination of this multi-step process, failure in wound healing stages can lead to chronic wounds. Several factors can lead to impaired wound healing including hypoxia, ischemia- reperfusion injury, bacterial colonization and altered collagen synthesis caused by systemic illness or chronic conditions including malnutrition and smoking, as well as local factors such as pressure, tissue edema and dehydration. Due to the imbalance between the production and degradation of collagen and growth factors, chronic wounds take a prolonged time to heal. Neutrophil infiltration to the wound site is regarded as an important factor for prolonged inflammation. Macrophages and neutrophils increase matrix metalloproteinase production leading to extracellular matrix protein degradation [27].

Recent studies have shown that natural-based or synthetic compounds possess notable advantages in terms of wound healing process modulation via Nrf2 signaling as detailed in further sections. Many Nrf2 activators have been reported to control oxidative stress by regulating the redox homeostasis, therefore leading to improved wound healing [12].

## 3. Gene Structure of Nrf2 Transcription Factor and Its Repressor Keap1

**Figure 1:**
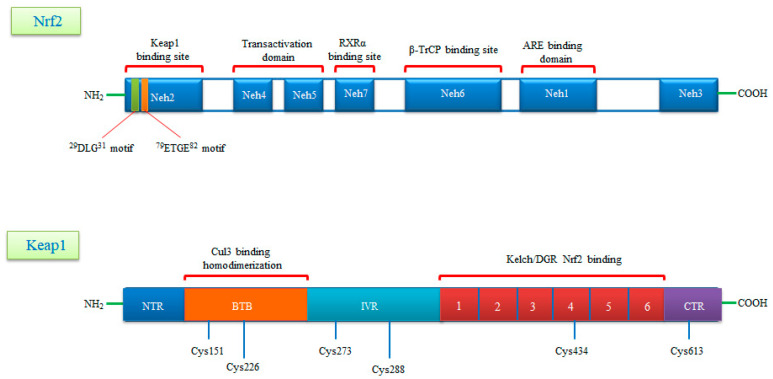
A schematic diagram of domain structures of Nrf2 and its repressor Keap1. Nrf2 consists of seven conserved domains known as Neh1-Neh7 and 605 amino acids. Neh1 interacts with small Maf (sMaf) proteins thanks to its Zip motif and thus binds to ARE sequences in DNA. The Neh2 domain contains two motifs called DLG and ETGE and interacts with the Keap1 molecule. The Neh4 and Neh5 domains are the CBP binding sites. Neh6 is a serine-rich domain that is required for TrCP binding and regulates Nrf2 stability. Neh7 is required to bind RXRα and inhibits ARE gene activity. Keap1, rich in cysteine residues, consists of five domains known as NTR, BTB, IVR, DGR, CTR and 624 amino acids. Keap1, rich in cysteine residues, consists of five domains known as NTR, BTB, IVR, DGR, CTR and 624 amino acids. The BTB domain provides homodimerization of Keap1 and is the binding site for Cullin3 (Cul3). The N-terminal region of the IVR domain mediates these functions and acts as a sensor for Nrf2 inducers. The Kelch/DGR domain inhibits the multiple ubiquitination of Nrf2 by mediating the binding of Nrf2 to the Neh2 domain.

Nrf2 is a basic leucine zipper (bZIP) transcription factor with Cap ‘n’ Collar (CNC) structure and interacts with the cysteine thiol groups of the protein Keap1 [28]. Keap1 acts as both an oxidative stress sensor and a regulator of the F-actin filament structure by virtue of its actin binding property. Nrf2 consists of seven functional Nrf2-ECh homology domains (Neh1-Neh7) [29]. The Neh1 domain provides the dimerization of Nrf2 with members of the musculoaponeurotic fibrosarcoma (Maf) protein and the DNA binding to the antioxidant response element (ARE) [30]. The Neh2 domain mediates the binding of Keap1 protein and Nrf2, and contains two different motifs, known as DLG and ETGE. The Neh3 domain is associated with stabilization of the protein, while the Neh4 domain is responsible for histone acetylation. The Neh4 and Neh5 domains are associated with transcriptional activation and promote the binding to the cAMP response element binding protein (CREBP). The Neh6 domain contains a number of serine residues that are recognized and subsequently phosphorylated by the protein glycogen synthase kinase 3 beta (GSK3β) leading to KEAP1-independent Nrf2 degradation. Lastly the Neh7 is an interaction domain with the nuclear receptor retinoic X receptor alpha (RXRa), that negatively controls Nrf2 activity. The Keap1 protein consists of five domains, an N-terminal region (NTR), a Tramtrack and Bric-à-Brac (BTB) domain, a central intervening region (IVR) with a nuclear export signal (NES) mediating the cytoplasmic localization of Keap1, six Kelch repeats and a C-terminal domain (CTR) [31]. The three domains, including, BTB, IVR and DGR domains, have binding sites. Highly reactive cysteine residues located within these domains (Cys151 and Cys226 in BTB, Cys273 and Cys288 in IVR) act as biochemical sensors of cellular stress and can be reversibly or irreversibly modified by ROS, RNS, H2S and other metabolites such as succinate or methylglyoxal. The DGR domain mediates the interaction between Keap1 and Nrf2 and directly binds to the ETGE motif of the Neh2 domain (Figure 1) [32,33].

## 4. Nrf2 Signaling: Mechanisms of Activation and Suppression

### 4.1. Canonical Activation of Nrf2

Under physiological conditions, Keap1 forms a homodimer that binds to the ETGE and DLG motifs in the Neh2 domain of Nrf2 causing its cytoplasmic retention. Keap1 is a substrate adaptor protein for the Cul3-based E3–ligase complex for ubiquitination and degradation by the ubiquitin proteosome system (UPS). It is estimated that under homeostatic conditions Nrf2 has a half-life of only 20 min, suggesting that its constant degradation is required to prevent unnecessary activation of the cytoprotective response [22,34,35].

By contrast, in the presence of cellular stress inducers such as ROS, xenobiotics and other electrophilic molecules, Nrf2 is unable to efficiently interact with the ubiquitin conjugating machinery due to a conformational change in the E3–ligase complex induced by the electrophilic modification of specific cysteines within Keap1 IVR or BTB domains [36]. Importantly, Keap1 is able to directly detect oxidative changes in the intracellular milieu thanks to the presence of redox-sensitive cysteine residues located within its regulatory domains that are subject to covalent modifications when Keap1 is exposed to electrophilic Nrf2-inducing chemicals such as hydrogen peroxide [37]. As a consequence, the inactive Keap1 molecules are progressively saturated by the pre-existing pool of Nrf2 while the neosynthesized Nrf2 molecules can escape Keap1 negative regulation and enter into the nucleus. Of note, Nrf2 can be activated by a number of different ARE inducers. Among them, hydrogen peroxide (H_2_O_2_) [38], NO [39], tertiary butylhydroquinone (tBHQ) [40], dimethyl fumarate (DMF) [41] as well as some phytochemicals (resveratrol, silymarin, sulforaphane, curcumin, cinnamic aldehyde etc.) [42] and bardoxolone methyl [43] are among the most well studied. They could protect the skin cells from UV damage and toxic chemicals. In addition, keratinocyte growth factor (KGF) and FGF, which are released by dermal fibroblasts after wound formation, also promote Nrf2 expression [44]. Heme released from heme proteins is required for Nrf2 stabilization in case of damage [45]. Additionally, Nrf2 can be activated through mitogen-activated protein kinase (MAPK) and protein kinase C (PKC) signaling pathways in response to oxidative stress [46].

**Figure 2:**
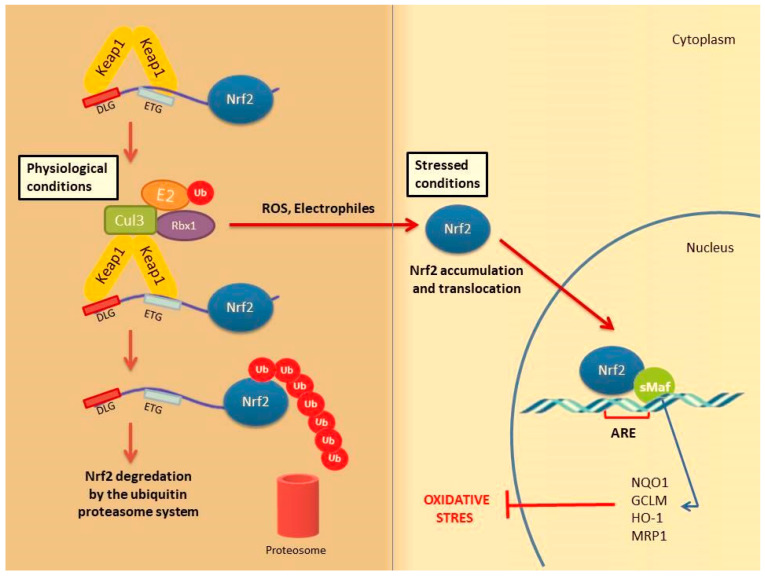
Nrf2 activation mechanism-canonical pathway. Under normal physiological conditions, Nrf2 forms a complex with the Keap1 protein Cul3 and Rbx, causing Nrf2 to be ubiquitinated and degraded by the ubiquitin proteasome system Under stress conditions, the Keap1 Cul3 complex is deactivated. This situation leads to the release and accumulation of Nrf2. Stable Nrf2 is translocated to the nucleus and binds to ARE together with small Maf proteins (sMaf). It ultimately activates antioxidant enzymes and cytoprotective proteins such as HO-1, NQO-1, GST. These enzymes support cellular defense by mediating the removal of ROS and cytotoxic electrophiles.

After Nrf2 is released from Keap1 and translocated into the nucleus, it is dimerized with the sMaf protein and binds to the ARE. It further regulates the expression of antioxidant enzymes and genes encoding cell protective proteins such as NAD(P) H: quinone oxidoreductase (NQO)-1, glutathione reductase (GSR), glutathione-S transferase (GST), glutathione peroxidase (GPx) superoxide dismutases 1–3 (SOD1–3), glutamate-cysteine ligase catalytic subunit (GCLC), peroxiredoxins (PRX), thioredoxins (TRX), catalase (CAT), heme oxygenase-1 (HO-1) and many others (Figure 2) [34,47].

### 4.2. Non-Canonical Activation of Nrf2: The Role of Autophagy

**Figure 3:**
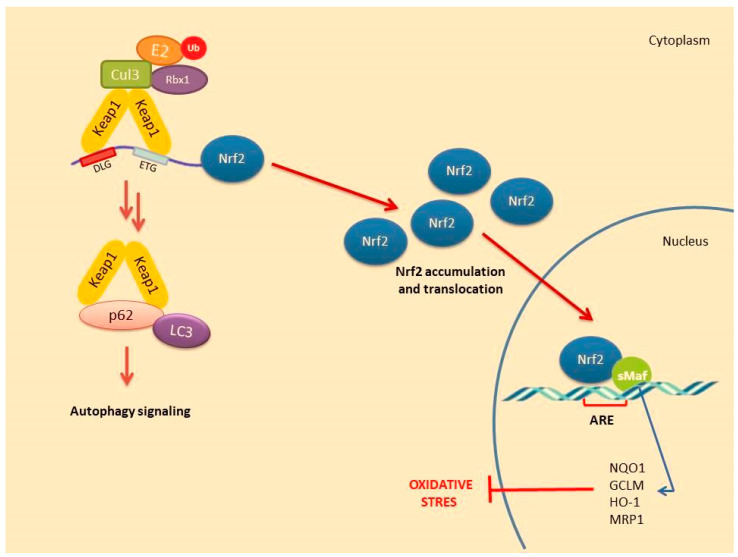
Nrf2 activation mechanism-canonical pathway associated with the induction of autophagy.

A number of alternative regulators of Nrf2 activation have been described and are collectively ascribed in the non-canonical pathway of Nrf2 activation. This pathway comprises a number of different proteins such as DPP3, PALB2, BRCA1, p62 and p21, possessing the ability to disrupt the formation of the Keap1-Nrf2 complex by preventing their reciprocal interaction through competitive binding, thus promoting Nrf2 stabilization and activation [48]. Possibly, the most well-studied mechanism of the non-canonical pathway is linked to autophagy. Autophagy is a highly conserved process involving the reuse of long-lived proteins and building blocks that are released as a result of the breakdown of damaged organelles. This conserved mechanism contributes to the restoration of cellular homeostasis and is activated in response to specific conditions such as nutrient scarcity, ROS overproduction, genomic instability, misfolded protein accumulation, organelle damage and microbial infection. Accumulating evidence also suggests that the autophagy mechanism can be triggered due to excessive ROS in cells. This mechanism allows misfolded or damaged proteins and damaged organelles to be broken down and reused for the cell purposes. Apart from this, it is also a vital defense mechanism in protecting against proteotoxicity induced by cellular redox stress [49]. Both autophagy and Nrf2/Keap1 have a protective role against oxidative stress (Figure 3).

In this pathway, p62 protein, which is an adapter protein that binds to ubiquitinated protein aggregates in autophagy and transmits it to autophagosomes. Upon serine phosphorylation within its 349-DPSTGE-354 motif, the p62 protein interacts with 3 arginines in the Kelch domain of Keap1, thus preventing or destabilizing Nrf2 binding. This further leads to Nrf2 stabilization and subsequent nuclear translocation inducing the transcription of several target genes [50]. Downmodulation of the p62 levels can thus restore the negative regulation of Keap1 and decrease Nrf2 stability [51,52,53].

## 5. Nrf2 Activation during Wound Healing

The main role of Nrf2 in wound healing is to detect the ROS accumulation in injured and inflamed tissues and to activate the antioxidant defense system [1]. Therefore, pharmacological induction of Nrf2 is an important therapeutic target in promoting healing after tissue damage and controlling repair-related inflammation [54]. Nrf2 pathway plays a protective role against oxidative stress through the expression of antioxidative enzymes during wound healing [55,56].

High ROS levels inhibit the proliferation of chondrocytes, stimulate apoptosis of osteocytes and increase bone resorption causing bone loss or delayed healing. Nrf2 transcription factor is expressed in many cell types including osteoblasts, osteocytes and osteoclasts. Lippross et al. showed that Nrf2 deficiency impairs normal fracture healing in Nrf2 knockout (KO, Nrf2^−/−^) mice [57]. Similarly, in Nrf2-KO mice, the absence of Nrf2 suppresses the expression of antioxidant enzymes in osteoblasts [58]. Although these results suggest a curative role of Nrf2 pathway during fracture healing, more studies are needed to better understand Nrf2 function in different bone cells [54]. 

Many growth factors such as basic FGF and insulin growth factor-1 (IGF-1) are responsible for modulating cellular responses after bone injury mediate fracture healing. Vascular endothelial growth factor (VEGF), one of the most crucial ones, promotes increased oxygenation in the injury site and initiates neovascularization. On the other hand, VEGF triggers osteoblast proliferation and migration [59]. Kweider et al. showed that VEGF activates Nrf2 in the BeWo cell line and enhances the levels of some antioxidant enzymes (thioredoxin, thioredoxin reductase and HO-1) [60]. However, this activation depends on the upstream induction of the ERK1/2 signal. These results confirm that Nrf2 promotes fracture healing by inducing antioxidant responses to protect cells from damage caused by ROS in bone damage.

During hemolysis, toxic-free heme is released by red blood cells. The conversion of heme to carbon monoxide and free iron is catalyzed by HO-1, which has an important immunomodulatory role. Indeed, it has been reported to have an anti-inflammatory effect by increasing the release of cytokines such as interleukin 10 (IL 10) and IL1β. Inhibition of free heme release by hemolysis is triggered by HO-1 expression caused by Nrf2 activation. In K565 cells (human pro-erytroid cells), hemin causes the ubiquitination of Keap1 and promotes Nrf2 stabilization. Therefore, targeting of the Nrf2/Keap1 system has been proposed to prevent heme toxicity [61].

Several reports have suggested that skin damage after UVB irradiation can lead to DNA modification and ROS formation [62]. In these cases, pharmacological activation of Nrf2 could be an effective way of reducing UVB cytotoxicity. In transgenic mice, endogenous Nrf2 prevents ROS damage caused by UVB and apoptosis of keratinocytes by inducing the activation of cytoprotective genes [63]. Topical application of Nrf2 activators sulforaphane (SFN) has been shown to protect the skin against acute UV toxicity [28]. In the bleomycin (BLM)-induced skin fibrous model, BLM was found to inhibit Nrf2 expression in the epidermis. However, Nrf2 deficiency in keratinocytes exacerbated skin fibrosis. This deficiency could also be correlated with the increased expression levels of Mcp-1, IL-6 and IL-8 [64]. Additionally, the formulation named RTA408 was shown to increase the expression of Nrf2 target genes and mediate re-epithelization [65]. Based on these evidence, Nrf2 pathway has a remarkable impact on healing of wounds and may be beneficial in the treatment of chronic wounds. Selected studies targeting Nrf2 activation against tissue damage in different experimental models are given in Table 1.

## 6. The Role of Nrf2 in Cell Proliferation, Apoptosis and Migration during Wound Repair

Nrf2 activation stimulates the proliferation and migration of epithelial cells during wound repair and inhibits apoptosis [66]. Loss of Nrf2 further slowed the epithelization process in the STZ-induced diabetic mouse model. This suggests that the deficiency of Nrf2 also impairs angiogenesis due to long-term inflammation and lack of neovascularization mediators. This is caused by the concomitant effects of increased apoptosis and oxidative damage accompanied by low TGF and high MMP levels. Nrf2 activation can be triggered by MMP9, transforming growth factor-β (TGF-β) and genes associated with migration and proliferation that promote wound healing in perilesional skin tissue taken from diabetic and ulcer patients [67].

**Figure 4:**
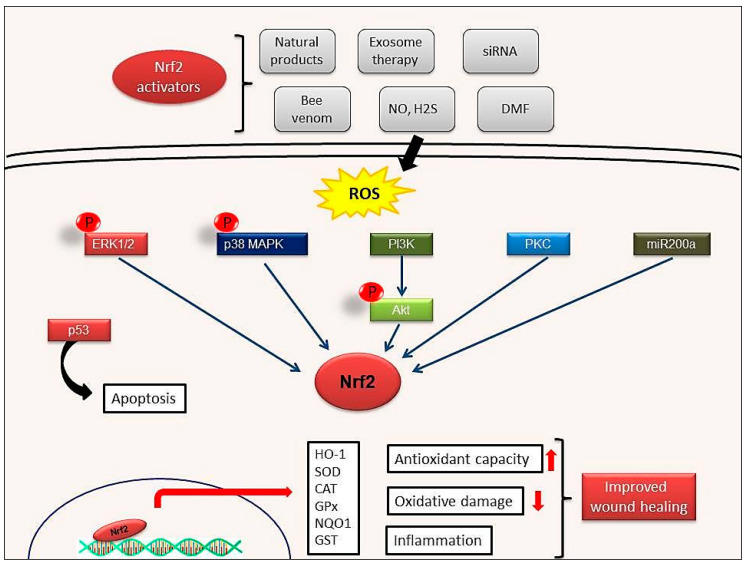
Possible wound healing mechanisms of Nrf2 activation. Activation of ERK1/2, p38MAPK, PI3K, PKC signaling pathways by Nrf2 activators. ERK1/2: Extracellular signal-regulated protein kinases 1 and 2; p38MAPK: p38 mitogen-activated protein kinases; PI3K: Phosphoinositide 3-kinase; PKC: Protein kinase C.

Endothelial dysfunction represents a further underlying deterrent to wound healing, especially in diabetes. A number of studies have suggested that bone-marrow derived endothelial progenitor cells (EPCs) contribute to postnatal neovascularization and vascular endothelial repair [68,69]. The number of circulating EPCs in humans [70] and in animals [71] with diabetes mellitus (DM) was reduced with a concomitant reduction in EPC functional ability (proliferation, colony formation, tube formation, self-renewal and mobilization) [72,73]. Recently, it has been reported that Nrf2 activation protects diabetic EPCs against the effects of oxidative stress and cell senescence, ameliorating the biological dysfunction of EPCs derived from mice with diabetes [74]. Thus, the targeting of endothelial dysfunction may represent another promising focus for future treatment of the diabetic wound with Nrf2 activators (Figure 4).

**Table 1:** Studies targeting Nrf2 activation against tissue damage.

Model	Injury	Method of Application	Treatment	Studied on	Results	Ref
in vivo	BLM-induced skin fibrosis	s.c. injection	-	Nrf2^−/−^ mice generated with Keratin 14-Cre/loxp system	Increased cytokines & chemokines expression (Mep-1, IL-6, IL-8)	[64]
in vivo	Corneal epithelial injury	i.m. injection	-	Nrf2^−/−^ mice	Induction of cell migration and inhibition of cell proliferation	[66]
in vivo	Endothelial cell injury	skin incision	-	Nrf2^−/−^ C57BL/6 mice	VEGF-induced proliferation ↓ Endothelial cell sprout formation ↓	[75]
in vitro	Retinal pericytes, astrocytes and endothelial cells	-	-	Blood-retinal barrier model	Increased IL-1β, IL-6, iNOS, Nox2 expression Activation of Nrf2 and HO-1	[76]
in vivo	Epidermal keratinocyte	i.p. injection (dorsal skin 10 mm)	*-*	*Lepr^db/db^* mice	Increased expression of *Nqo1* and *Sod2* genes due to impaired Nrf2 activity	[77]
in vivo	Nonhealing skin ulcers	i.p. injection	-	Nrf2^−/−^ C57BL/6 mice Perilesional skin tissue samples from diabetic patients	Increasing proliferation and migration, decreasing apoptosis Upregulation of TGF-β1 and downregulation of MMP9	[78]
in vitro	Endothelial dysfunction	-	-	Primary human coronary arterial endothelial cells (CAECs)	Impaired angiogenic processes (including proliferation, adhesion, migration and ability to form capillary-like structures)	[79]
in vivo	Chronic venous insufficiency-related wound injury	i.p. injection	RTA 408 (Omaveloxolone)	C57BL/6 mice	Expression of antioxidant mediators	[80]
in vivo	Bed wounds	s.c. injection (dorsal skin 12 mm)	ESC-Exos treatment	C57BL/6 mice skin aging model	Nrf2 activation, improved skin aging and downregulation of Keap1 by miR-200a	[81]
in vivo	Retina injury	intravitreally injection	MIND4-17	BALB/C mice	Activation of Nrf2 and reduced disfunction of retina Preventing apoptosis caused by high glucose	[82]
in vivo	Atherosclerotic lesions	i.p. injection	tBHQ	apoE^−/−^ mice	Increased expression of HMOX1, SOD1 and CAT Upregulation of autophagy-related genes (BECN1, SQSTM1/p2, ATG5/7)	[83]
in vivo	Impaired wound healing	i.p. injection	DMF	Wistar rats	Upregulation of Nqo1 and HO-1 expression Downregulation of IL1β, IL-6 and MCP1	[84]
in vivo	Impaired wound healing	i.p. injection (1.5 mm dorsal skin)	LPS-Exos (500 µg/mL –1 mg/mL, 21 day)	Sprague Dawley rats	Increased expression of Nrf2, HO-1 and Nqo1 genes Decreased expression of proinflammatory cytokines (IL-6, IL-1β, TNF-α) and MMP9	[85]
in vivo	Impaired wound healing	i.p. injection (8 mm dorsal skin)	PCB2 treatment (10 mg/kg daily)	C57BL/6 mice	Promoting cell survival and migration Decreased oxidative stress	[86]
in vitro in vivo	Cutaneous wound	i.p. injection (10 mm dorsal skin)	siKeap1	3T3 cells (added siRNA-liposomal complex) *Lepr^db/db^* mice	Redox homeostasis	[87]

i.m.: intramuscular; i.p.: intraperitoneal; s.c.: subcutaneous.

Tumor suppressor protein p53, one of the most well-known apoptosis inducers, is also one of the sensors of oxidative stress. It also supports cell survival by acting as an antioxidant during cellular stress. p21 protein, another crucial protein participating to cell cycle arrest and apoptosis induction, can affect the transcription of ARE genes. Schmidt et al. showed that cold plasma promotes Nrf2- and p53-mediated granulation and reepithelization in the skin of SKH1 mice. Levels of proinflammatory cytokines IL-6 or TNFα and angiogenetic factors such as FGF, KGF, VEGF and COX2 increase in the early stages of wound healing and this increase depends on Nrf2 signal activation [4]. This observation also indicates that mild inflammation is necessary to trigger wound healing [56].

Recent studies have made significant contributions to elucidate the relationship between the Nrf2/Keap1 signaling pathway and wound healing. However, some points still need to be clarified. In particular, we know very little about the interaction and regulatory mechanisms between signal pathways that trigger autophagy and the Nrf2 signaling. In case of tissue damage and severe ROS production, Nrf2 mediates the prevention of apoptosis of keratinocytes caused by the activation of cytoprotective genes. It is well accepted that Nrf2 acts as a defense signal in the case of oxidative stress and plays a cell protective role by reducing ROS. However, there are studies showing that continuous activation of Nrf2 can also trigger tumorigenesis. Given the dualistic role of Nrf2, many factors such as the contribution of additional signaling pathways, activators and suppressors or even inflammatory mediators should be taken into account to understand how the Nrf2 pathway regulates the wound healing activity. However, formulations to be developed using Nrf2 activators in acute or chronic wounds are seen as a promising treatment.

## 7. Nrf2 Induction of Cytoprotective Genes and Nrf2 Dependent Therapeutic Regimens in Wound Healing

Besides the harmful effects caused by their excessive or prolonged production, ROS confer protection against pathogens and prevent infections in the wound area when present at low-moderate levels. Importantly, ROS are produced by both epithelial and migrating reparative cells (keratinocytes, macrophages, neutrophils). However, long-term inflammation in unhealed chronic wounds and oxidative damage caused by excessive production of ROS impairs cell proliferation and leads to apoptosis [55].

It is known that the wounds of diabetic individuals heal more slowly, while their neovascularization is largely impaired and even wounds can worsen much faster than nondiabetic counterparts. The main reason behind these alterations is strictly related to the existence of more severe oxidative stress conditions than those experienced by normoglycemic individuals. In vitro cell experiments showed that hyperglycemia treatment suppresses Nrf2 activation, resulting in oxidative stress with decreased expression of antioxidant genes, together with increased secretion of proinflammatory cytokines [88]. The ARE is present in the regulatory regions of various genes encoding cytoprotective enzymes. ARE is activated by Nrf2 binding and is a promising therapeutic target to concomitantly stimulate wound healing via inflammation and reduce the burden of oxidative stress in diabetic patients [89].

Nrf2 activators have been reported as contributing to the fight against several pathologic conditions, such as diabetes, Alzheimer disease, cardiovascular disease, diabetic foot ulcer and diabetic nephropathy. Cui et al. demonstrated that SFN treatment as NRF activator increased renal Nrf2 expression, prevented diabetic nephropathy and thereby attenuated both oxidative damage and fibrosis in a diabetic mouse model [90]. tBHQ is another Nrf2 activator with strong oxidizing properties that binds to the cysteine residues of Keap1, causing Nrf2 stabilization [91]. On the other hand, SFN treatment leads to nuclear translocation and accumulation of Nrf2 and increases HO-1 and NQO-1 expressions in the diabetic retinopathy [84]. In STZ-induced diabetic rats, tBHQ treatment increases the expression of Nrf2 in macrophages and vascular smooth muscle cells in atherosclerotic lesions [92]. It is predicted that tBHQ can be an atheroprotective approach with promising applications in diabetes. In another study investigating the wound healing activity of syringic acid (SA), an Nrf2 activator, SA, was reported to reduce ischemia damage by triggering Nrf2 activation [93]. Ren et al. confirmed that SA increases wound healing in diabetic rats. On the other hand, SA decreases the expression of Keap1 and increases the expression of antioxidative enzymes (SOD, CAT, GPx, GST, GR) [11]. However, after Nrf2 activator DMF treatment, oxidative stress decreased and diabetic wound healing increased significantly, while Nrf2 inhibitor ML385 mimicked the effect of diabetes [84]. In a recent study using a diabetic rat model, the wound area has been significantly improved with the use of exosomes of fat-derived stem cells (ADSCs) causing overexpression of Nrf2 [94]. However, a change in the Nrf2 gene might also render the wound area more vulnerable to complications of diabetes (such as nephropathy and retinopathy) [95]. There is a recent study investigating whether wound healing was triggered by lipopolysaccharide stimulated macrophage exosomes (LPS-Exos) in STZ-induced hyperglycemia rats. The results showed that LPS-Exos decreased ROS and MDA levels in the scar tissue of rats, as well as increased the production of SOD and GSH-Px. It also activates the Nrf2/HO-1 signaling. This effect of LPS-Exos can be considered as a novel pharmacological agent that can repair wound healing in diabetic rats [85].

In the type I diabetic mouse model, the expression of collagen and p-defensin-2 genes is decreased compared to the control. The study investigating the effect of bee venom treatment on diabetic wound healing, showed that bee venom supports epithelial cell proliferation and migration by increasing Nrf2 expression [2]. In a study investigating the wound healing activity of plumbagin (a bioactive naphthoquinone isolated from *Plumbago zeylanica* L. roots), it was observed that plumbagin increased epithelization and collagen deposition. At the same time, plumbagin decreased the levels of inflammatory cytokines tumor necrosis factor α (TNFα), IL-6 and IL-1β in diabetic rats [96]. Therefore, plumbagin, with both anti-diabetic and anti-inflammatory effects, can be used in the pharmacological activation of Nrf2 in the wound healing model. In another study evaluating the wound healing activity of neferine, an alkaloid obtained from the seeds of the lotus plant was investigated in a diabetic mouse wound model (10mm full-thickness wound on the dorsal skin). After the topical application of neferine (10% and 20% for 14 d), the level of ECM-related genes such as collagen-1, TGF-β and the increase in the activities of antioxidant enzymes such as SOD, CAT and GST accelerates wound healing [97].

Additionally, it is already known that molecules such as NO and H2S contribute to wound healing. However, the role of H2S in re-epithelization during wound healing is not as clear as it is for NO. A recent study suggests that H2S is effective in controlling wound keratinocytes [14]. It was initially hypothesized that H2S-mediated Nrf2 regulation did not affect CK10 expression because the CK10 promoter did not contain the Nrf2 binding site. However, a binding site was later found in the CK10 promoter. Accordingly, H2S triggers the expression of cytokeratin 10 (CK10) in keratinocytes. However, additional studies are needed to clarify the mechanisms of the H2S-mediated Nrf2 regulation of CK10 expression. Lastly, Rabbani et al. found an effective way to successfully deliver the targeted siRNA against Keap1 by developing a liposome and protein hybrid nanoparticle formulation (lipoproteoplex (LPP)) in the Male diabetic Lepr^db/db^ mouse model. As it is known, siRNAs are designed to target and degrade specific mRNA transcripts. This practice has shown promising results in enhancing diabetic wound regeneration and improving redox homeostasis in the wound area [98]. Although the siRNA delivery system can be applied for diabetic wound treatment, additional clinical studies are required. Because 21–23 bp siRNA is susceptible to degradation by nucleases, this might represent a strong limitation to the effective delivery of a specific siRNA into the cell.

The studies mentioned so far have been oriented on the exploitation of the protective role of Nrf2 in cellular stress conditions such as ROS, electrophiles, and proteotoxic stress. However, according to some studies, Nrf2 activation can also potentially lead to undesired side effects. For example, prolonged activation of Nrf2 through the deletion of Keap1 was found to cause hyperkeratosis in murine skin [33]. Schäfer et al. similarly determined that long-term activation of Nrf2 in keratinocytes caused seborrhea in mice due to the upregulation of the growth factor epigen, which is considered a new Nrf2 target [99]. On the other hand, accumulating evidence also indicate that Nrf2 activation may even support tumorigenesis and malignant progression, thereby promoting unrestrained cell proliferation, cancer cells adaptation and therapy resistance in a number of solid and malignant tumors [100].

Since Nrf2 activation is a potential approach for the clinical management of diabetic wound healing, chemical compounds could accelerate wound healing in Nrf2-dependent manner (Table 2). DMF, a well-proven potent Nrf2 activator, demonstrated attenuation of inflammation and ROS production and thereby accelerated wound closure in a diabetic mouse model [101]. An advantage of DMF is its approval in clinical use for treatment in multiple sclerosis, which proves its safe nature. Similarly, other most well-known potent Nrf2 activators, sulforaphane and cinnamaldehyde, were shown to be effective against the diabetic wound healing in a mouse model [102]. Ketoconazole, an antifungal azole group compound, accelerates wound healing, which is directly correlated with the increase in the transcriptional Nrf2 activity and reduced inflammatory response [103].

Nrf2 activation in fibroblasts is also induced by many natural compounds including polyphenols, such as curcumin, epigallocatechin-3-gallate (EGCG) or apomorphine, and flavone derivatives as well as many bioactive components of pepper beetle, brassica plants and walnut sprouts extracts [120]. Similarly, procyanidin B2, one of the major bioactive components of procyanidins promotes wound healing in diabetic mice in a Nrf2-dependent manner [86]. Curcumin, a potent Nrf2 activator, accelerated the cutaneous wound healing in streptozotocin-induced diabetic rat model [121]. Treatment with Huangbai liniment, a traditional Chinese medicine, demonstrated acceleration of the wound closure and increase in the generation of extracellular matrix in a diabetic rat model via Nrf2 activation [122]. Neferine, an alkaloid present in lotus, significantly accelerates wound closure rate, followed by a decrease in the period of re-epitalization with a higher amount of collagen via Nrf2 signaling pathway [98]. On the other hand, berberine treatment remarkably accelerated wound healing and increased extracellular matrix synthesis via Nrf2 pathway [123]. Another Nrf2 activator, carnosol, significantly increased wound healing capacity in retinal epithelial cells [107]. In vitro and in vivo studies also suggest that treatment with the alkyl catechols, 4-ethyl catechol and 4-vinyl catechol, potent Nrf2 cofactors, are used traditionally for diabetic wound healing [35].

## 8. Conclusions

Despite significant advances in therapeutic strategies used to accelerate the healing of wounds, there is still no practical cure for chronic wounds. In recent years, there has been a growing interest in antioxidant capacity of Nrf2. Several researchers have examined Nrf2-activating compounds to prevent and treat chronic inflammatory and degenerative disorders. It has been revealed that Nrf2-inducing bioactive compounds that improve the wound healing process may be a promising therapeutic approach for treating chronic wounds. However, it is an important issue to investigate the safety of Nrf2 activators in specific animal models and clinical trials before their applications in the medical practice.

## Figures and Tables

**Table 2:** Recent studies on natural products inducing Nrf2 activation.

Compound/Extract/Material	Injury	Model	Studied on	Site of Action	Ref
Apigenin	Age-related macular degeneration	in vitro	Human retinal epithelial cell line (ARPE-19)	Antioxidant properties depending on Nrf2 activation Upregulation of SOD, CAT, GSH-Px, T-AOC Downregulation of ROS	[104]
Apocarotenoid (bixin)	Radiation-induced dermatitis	in vivo	SHK1 mice (Dorsal skin exposed to UV radiation)	Prevention of DNA damage and oxidative stress	[105]
Bee venom	-	in vivo	BALB/c mice	Enhanced wound by increasing collagen and BD-2 expression Enhanced Ang-1/Tie-2 downstream signaling	[2]
Catexanes	Oxidative stress	in vitro	Human gastric epithelial cells (AGS)	Reduced Keap-1 expression and induced NQO1 expression	[106]
Topical application of carnosol	Corneal epithelial injury	in vivo	Sprague-Dawley rats	Oxidative stress responses	[107]
Curcumin	-	in vitro	Porcine renal epithelial proximal tubule cells (LLC-PK1) and rat kidney epithelial cells (NRK-52E)	Increased HO-1 protein expression and heme oxygenase activity Activation of the Nrf2/ARE pathway Phosphorylation by p38 MAPK	[108]
Dihydromyricetin	Vascular endothelial cell injury	in vitro	Human umbilical vein endothelial cells (HUVEC)	Activation of the Akt and ERK1/2 pathways	[109]
EGCG	Oxidative stress	in vitro	Bovine aortic endothelial cells (BAECs)	Upregulation of HO-1 Activation of Akt and ERK1/2 signaling	[110]
Grape seed proanthocyanidin extract	Diabetic retinal function	in vivo	Wistar rats	Increased SOD and GSH-Px activity levels	[111]
Hydroxytyrosol	-	in vitro	Human retinal pigment epithelial cells (ARPE-19)	Overexpression of Nrf2 and increased GSH content Activation of the PI3/Akt and mTOR/p70S6-kinase pathways Upregulation of p62/autophagy	[112]
*Lycium barbarum* polysaccharides	Retina injury	in vivo	i.p. injection of ZnPP	Accumulation of Nrf2 and HO-1 expression	[113]
Methyleugenol	Oxidative stress	in vitro	Murine macrophage cells (RAW 264.7 and J774A.1)	Activation of the AMPK/GSK3β and ERK-Nrf2 signaling pathways	[114]
Olive oil-based diet	IR injury	in vivo	External application of magnet disks (dorsal skin)	Decreased COX-2 and increased NO synthase-2, Nrf2 and collagen type 1 protein expression	[115]
Resveratrol	Endothelial dysfunction	in vitro	Primary human coronary arterial endothelial cells (CAECs)	Upregulation of NAD(P)H:quinone oxidoreductase 1, γ-glutamylcysteine synthetase, and HO-1	[116]
Rutin	Diabetic neuropathy	in vivo	Sprague–Dawley rats	Decreased caspase-3 expression increased hydrogen sulfide (H2S) level	[117]
α-Tocopherol	Retina injury	in vitro	Human retinal pigment epithelial cells (ARPE-19)	Activation of Keap1/Nrf2 signaling Expression of HO-1, GST, SOD enzymes	[118]
Withaferin A	-	in vitro	Human umbilical vein endothelial cells (HUVECs) and endothelial cell line (EA.hy926)	Increased expression of HO-1 Direct interaction with Keap1	[119]

## Data Availability

Not applicable.

## Abbreviations

ADSCs	adipose-derived stem cells
ARE	antioxidant response element
BTB	broad-complex, tramtrack and bric a brac
bZIP	basic leucine zipper
CAT	catalase
CK10	cytokeratin 10
CNC	cap ‘n’ collar
COX2	cyclooxygenase 2
CREBP	cAMP response element binding protein
Cul3	cullin3
DGR	double glycine repeat
DMF	dimethyl fumarate
EGCG	epigallocatechin-3-gallate
EGFR	epidermal growth factor receptor
ECM	Extracellular matrix
FGF	fibroblast growth factor
GCLC	glutamate-cysteine ligase catalytic subunit
GPx	glutathione peroxidase
GSR	glutathione reductase
GST	glutathione S-transferase
H2S	hydrogen sulfide
H2O2	hydrogen peroxide
HO-1	heme oxygenase 1
IL-10	interleukin 10
IL1β	interleukin 1β
IL-6	interleukin 6
IVR	intervening region
Keap1	kelch-like ECH-associated protein 1
KGF	keratinocyte growth factor
Maf	musculoaponeurotic fibrosarcoma
MAPK	mitogen-activated protein kinases
MMP	matrix metalloproteinases
Neh	Nrf2-ECh homology
NF-κB	nuclear factor kappa-light-chain-enhancer of activated B cells
NO	nitric oxide
NQO-1	NAD(P)H:quinone Oxidoreductase 1
Nrf2	nuclear factor E2 p45-related factor 2
PKC	protein kinase C
ROS	reactive oxygene species
RXRα	retinoid X receptor α
SA	syringic acid
SFN	sulforaphane
siRNA	small interfering RNA
SOD1-3	superoxide dismutase1-3
STZ	streptozotocin
tBHQ	tertiary butylhydroquinone
TF	transcription factor
TGF-β	transforming growth factor-β
TNFα	tumor necrosis factor α
TrCP	transducin repeat-containing protein
TRX	thioredoxin
UPS	ubiquitin proteasome system
VEGF	vascular endothelial growth factor
